# Aggregation of Cystatin C Changes Its Inhibitory Functions on Protease Activities and Amyloid β Fibril Formation

**DOI:** 10.3390/ijms22189682

**Published:** 2021-09-07

**Authors:** Abdullah Md. Sheikh, Yasuko Wada, Shatera Tabassum, Satoshi Inagaki, Shingo Mitaki, Shozo Yano, Atsushi Nagai

**Affiliations:** 1 Department of Laboratory Medicine, Faculty of Medicine, Shimane University, 89-1 Enya Cho, Izumo 693-8501, Japan; abdullah@med.shimane-u.ac.jp (A.M.S.); tabassum@med.shimane-u.ac.jp (S.T.); syano@med.shimane-u.ac.jp (S.Y.); 2 Department of Neurology, Faculty of Medicine, Shimane University, 89-1 Enya Cho, Izumo 693-8501, Japan; wadayasu@med.shimane-u.ac.jp (Y.W.); inasato061009@yahool.co.jp (S.I.); shingomi@med.shimane-u.ac.jp (S.M.)

**Keywords:** Cystatin C, aggregation, cathepsin activity, Amyloid β, neurodegeneration

## Abstract

Cystatin C (CST3) is an endogenous cysteine protease inhibitor, which is implicated in cerebral amyloid angiopathy (CAA). In CAA, CST3 is found to be aggregated. The purpose of this study is to investigate whether this aggregation could alter the activity of the protein relevant to the molecular pathology of CAA. A system of CST3 protein aggregation was established, and the aggregated protein was characterized. The results showed that CST3 aggregated both at 80 °C without agitation, and at 37 °C with agitation in a time-dependent manner. However, the levels of aggregation were high and appeared earlier at 80 °C. Dot-blot immunoassay for oligomers revealed that CST3 could make oligomeric aggregates at the 37 °C condition. Electron microscopy showed that CST3 could make short fibrillary aggregates at 37 °C. Cathepsin B activity assay demonstrated that aggregated CST3 inhibited the enzyme activity less efficiently at pH 5.5. At 7.4 pH, it lost the inhibitory properties almost completely. In addition, aggregated CST3 did not inhibit Aβ_1-40_ fibril formation, rather, it slightly increased it. CST3 immunocytochemistry showed that the protein was positive both in monomeric and aggregated CST3-treated neuronal culture. However, His6 immunocytochemistry revealed that the internalization of exogenous recombinant CST3 by an astrocytoma cell culture was higher when the protein was aggregated compared to its monomeric form. Finally, MTT cell viability assay showed that the aggregated form of CST3 was more toxic than the monomeric form. Thus, our results suggest that aggregation may result in a loss-of-function phenotype of CST3, which is toxic and responsible for cellular degeneration.

## 1. Introduction

Cystatin C (CST3) is a cysteine protease inhibitor, which is expressed ubiquitously in every type of cell [1]. It contains 120 amino acids, and its molecular weight is 13 KDa [1]. CST3 is found to be localized in lysosomes [2,3], where it plays an important role in controlling the functions of the organelle by regulating the activity of cysteine protease enzymes including cathepsin B, H, and L [4,5]. It is also localized in the endoplasmic reticulum and Golgi complex, indicating it as a secreted protein [1,6]. In the extracellular space, it is believed to neutralize the activities of proteases that are often released or leaked from the lysosomes of dying or diseased cells [7,8]. The expression of CST3 in the brain is high [9]. In addition, its CSF concentration is about five times higher than that of plasma [10]. Such findings indicate its important role in brain homeostasis. In neuroinflammatory conditions, microglia are demonstrated to secrete cysteine proteases in the extracellular space [11]. Here, CST3 can inhibit the activities of such cysteine proteases, and thereby regulates the neuroinflammatory conditions. Hence, the dysregulation of such inhibitory effects of CST3 could alter the neuroinflammatory conditions leading to neurodegeneration.

Proper functioning proteins are required for proper functioning cells, tissues, or organs. After release from the ribosome, the unfolded polypeptide chain must adopt a proper three-dimensional structure to perform its function. To perform their functions, sometimes proteins undergo self-assembly such as dimerization or polymerization or combine with other proteins to make a functioning complex for the cells. However, in pathological aggregation, namely, amyloid aggregates, the proteins aggregate in a way that changes their functions [12]. Such pathological aggregation can be the core component of several diseases, including neurodegenerative diseases. For example, amyloid β (Aβ) peptide and Tau aggregation are implicated in Alzheimer’s disease, and α-synuclein aggregation is related to Parkinson’s disease [13,14]. CST3 is also shown to be aggregated in diseases such as cerebral amyloid angiopathy (CAA), where it is deposited on the vessel walls along with Aβ peptide, leading to brain hemorrhage and stroke [15]. In the case of inherited CAA, a mutation (L68Q) in *CST3* was identified [16]. L68Q mutation increases its aggregation properties [16,17]. CST3 can bind to Aβ peptide and inhibits its aggregation [18]. It is possible that aggregated CST3 still can bind to Aβ peptide but loses its amyloid inhibition properties, resulting in their co-deposition on the vessels. In addition, deposition might change its protease inhibitory properties and alter the balance of protease activity, which crucially affect the integrity of the vessel wall [7]. Furthermore, in sporadic CAA cases, CST3 without any mutation was found to be deposited on the vessel walls along with Aβ, indicating the importance of aggregation for the disease pathology [19].

The misfolding or aggregation of a protein may result in a loss-of-function, or a gain-of-function phenotype, which could alter the cellular functions crucial for the cell viability. In a previous study, we showed that CST3 is localized in Bunina bodies, the inclusion bodies found in the motor neurons of ALS spinal cords [20]. In addition, previous studies showed that the exogenous administration of CST3 can make Bunina body-like intracellular aggregates in the motor neurons of ALS model mice [21]. Since it is found in the inclusion bodies, it is possible that CST3 is aggregated here, and that makes it resistant to degradation. However, it is not known how such aggregation changes the activity of the protein. In a study, it is shown that the CST3 dimer, made by domain swapping, loses its protease inhibitory activity [22]. In CAA or ALS, deposited CST3 could be not only dimers, but also be higher order aggregates. Hence, it is important to characterize CST3 aggregation in terms of its structural changes, conditions that induce aggregation, and changes of activities, especially cathepsin inhibitory activity. Such information could be important for a better understanding of disease pathology such as CAA. Therefore, in this study, we aimed to investigate whether the aggregation of CST3 could change some of its activity. 

## 2. Results

**Figure 1:**
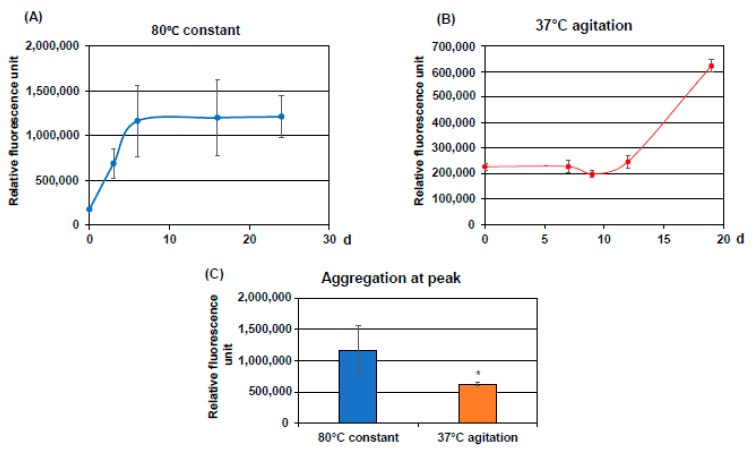
Evaluation of CST3 aggregates: Recombinant CST3 was incubated at 80 °C without agitation or at 37 °C with agitation for indicated times. Aggregation of CST3 was evaluated using an aggregation assay kit, as described in the Materials and Methods. The results were expressed as the average ± SD of an arbitrary fluorescence unit. (**A**) shows the fluorescence when CST3 was incubated at 80 °C, and (**B**) is the fluorescence when CST3 was incubated at 37 °C. (**C**) shows the aggregated protein levels at the plateau. Statistical significance is denoted as follows.

*Establishment of a system of CST3 aggregation***:** Recombinant CST3 (1.3 mg/mL) was incubated at 80 °C without agitation or at 37 °C with agitation. Periodically, CST3 was withdrawn from the incubated samples and the protein aggregation was measured. The results are presented in Figure 1. In the case of the 80 °C condition, the aggregation of protein started from 3 days of incubation and reached a plateau at 6 days (Figure 1A). When CST3 was incubated at 37 °C with agitation, protein aggregation was not observed until 12 days. At 19 days, protein aggregation was increased in this condition, however, that was not as high as at 80 °C without agitation (Figure 1B,C).

**Figure 2:**
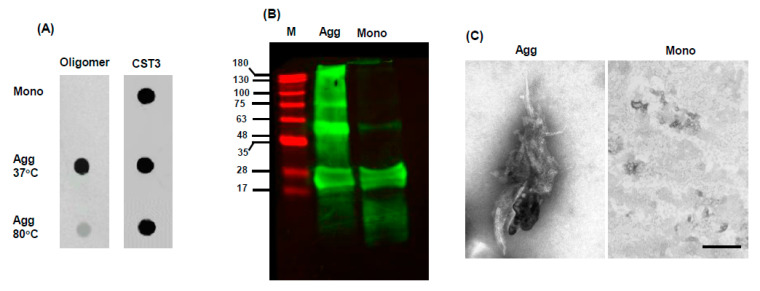
Characterization of CST3 aggregates: For characterization, aggregated CST3 was evaluated by dot-blot immunoassay. After aggregation, the samples were spotted on a nitrocellulose membrane and blotted with an oligomer conformation-specific antibody. (**A**) shows a representative dot-blot immunoassay data. For loading control, the same membrane was blotted with anti-CST3 IgG. The samples incubated at 37 °C were further evaluated by Western blotting and electron microscopy. Aggregated and monomer samples were separated using a 15% polyacrylamide gel, transferred to a PVDF membrane, and blotted with anti-CST3 IgG. Representative Western blotting data is shown in (**B**). In (**C**), electron microscopy photomicrographs of CST3 in monomeric and aggregated forms are shown. Mono = CST3 monomer, Agg = CST3 aggregated at 37 °C. Scale bar = 200 nm.

*Characterization aggregated CST3:* For characterization, we performed a dot-blot immunoassay using an oligomer conformation-specific antibody. The results showed that the oligomeric CST3 levels were increased in the 37 °C incubation condition compared to that of the 80 °C condition (Figure 2A). Subsequently, the aggregates made at 37 °C were evaluated by Western blotting and electron microscopy. The Western blotting results revealed that CST3 aggregated to several oligomeric species, starting from dimer (Figure 2B). In this condition, CST3 also adopted a fibrillary structure, which was short and broad in morphology (Figure 2C).

**Figure 3:**
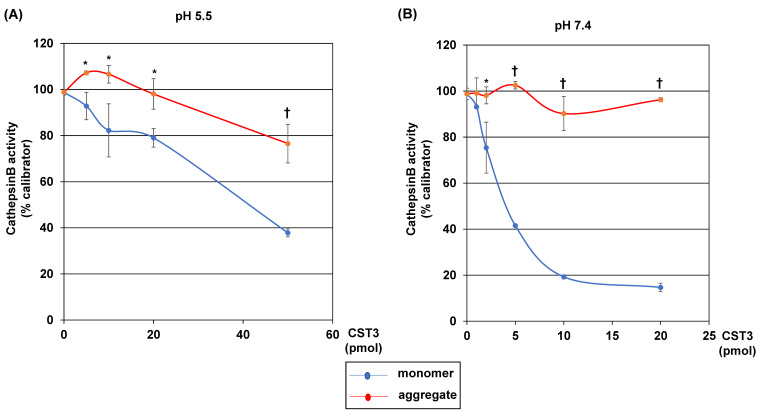
Effects of CST3 aggregation on cathepsin B activity. Cathepsin B activity was measured in the absence or presence of indicated concentrations of non-aggregated or aggregated CST3 at pH 5.5 and 7.4, as described in the Materials and Methods. Cathepsin B activities at pH 5.5 and 7.4 are shown in (**A**,**B**), respectively. The data of cathepsin B activity presented here as the average ± SD of ‘%calibrator’, where cathepsin B activity of one sample in the absence of CST3 was considered as such. Statistical significance is denoted as follows: * *p* < 0.01 vs. corresponding CST3 monomer, and ^†^ *p* < 0.001 vs. corresponding CST3 monomer conditions.

*Effects of CST3 aggregation on cathepsin B activity*: Next, we checked if aggregation changes the function of CST3. Since it is an endogenous cysteine protease inhibitor, we investigated if aggregation changes its inhibitory effects on the activity of a cysteine protease cathepsin B. The cathepsin B activity was measured at pH 5.5 and 7.4 to mimic the conditions of lysosome and extracellular space, respectively. The results showed that the CST3 monomeric form dose-dependently inhibited cathepsin B activity at both the pH 5.5 and 7.4 conditions (Figure 3A,B). In the case of the CST3 aggregated form, the ability of inhibition was decreased at pH 5.5 (Figure 3A). Importantly, at pH 7.4, the aggregated CST3 did not show any inhibitory effects on the cathepsin B activity (Figure 3B).

**Figure 4:**
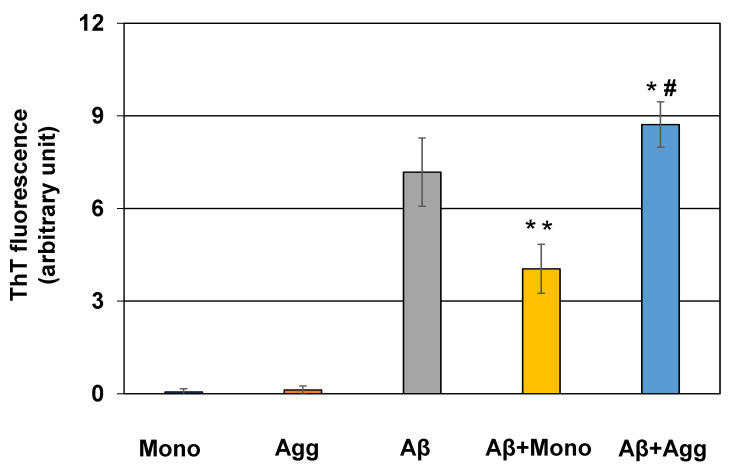
Effects of CST3 aggregation on Aβ_1-40_ fibril formation. Aβ_1-40_ (25 μM) was incubated in the absence or presence of non-aggregated or aggregated CST3 for 48 h, as described in the Materials and Methods. Amyloid fibrils formed after incubation were measured by ThT fluorescence assay. Normalized fluorescence emission values are shown here, and the data are presented as the mean ± SD of at least three independent experiments. Mono = CST3 monomer, Agg = CST3 aggregated at 37 °C. Statistical significance is denoted as follows: * *p* < 0.05 vs. Aβ_1-40_, ** *p* < 0.005 vs. Aβ_1-40_, ^#^ *p* < 0.0001 vs. Aβ_1-40_ and CST3 monomer condition.

*Effects of CST3 aggregation on Aβ fibril formation:* Previously, it was demonstrated that CST3 inhibits Aβ aggregation and protects the neurons from aggregation-induced toxicity [17]. Hence, we checked if aggregation changes such a function of CST3. The incubation of a CST3 monomer with Aβ_1-40_ decreased its total fibril levels, as revealed by ThT fluorescence assay. Interestingly, when Aβ_1-40_ was incubated with an aggregated form of CST3, the total fibril levels were not decreased; rather, they slightly but significantly increased (Figure 4). We did not detect an appreciable amount of amyloid fibrils when a CST3 monomer or aggregated forms were incubated alone.

**Figure 5:**
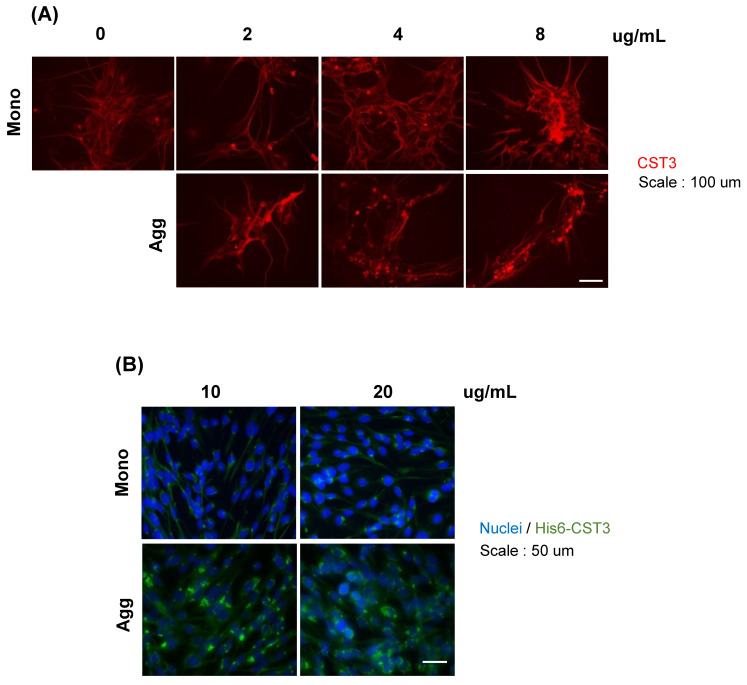
CST3 internalization in neuronal cell culture. (**A**) Indicated concentrations of recombinant CST3 in non-aggregated or aggregated form were added to a mouse neuronal line and cultured for 4 days. Intracellular CST3 was detected by immunocytochemistry using anti-CST3 IgG (red). (**B**) A human astrocyte cell line (CCF-STTG1) culture was incubated with indicated concentrations of His6-tagged recombinant non-aggregated and aggregated CST3 for 48 h. After culture, the presence of intracellular CST3 was evaluated by His6 immunocytochemistry (green). Nuclei were stained with Hoechst (blue) Mono = CST3 monomer; Agg = aggregated CST3 at 37 °C.

*Cellular internalization of CST3:* Then, we investigated the cellular internalization of CST3 by immunocytochemistry. After 4 days of incubation with monomeric or aggregated protein, the immunocytochemistry of a neuronal cell culture (NMW7) showed that in both the monomeric and aggregated condition, CST3 was positive inside the cells (Figure 5A). In addition, low levels of immunopositive signals were observed in the neuronal cells without CST3 treatment. Since neuronal cells express CST3, to see the internalization and accumulation of exogenous His6-tagged recombinant CST3 inside the cells, His6 immunocytochemistry was employed. Here, we used an astrocytoma cell line (CCF-STTG1) because of the low levels of endogenous CST3 expression [23]. The results showed that when the cells were incubated with a monomeric form of CST3, His6 was difficult to detect inside the cells. Conversely, when the cells were incubated with an aggregate CST3, most of the cells were found to be His6 positive (Figure 5B).

**Figure 6:**
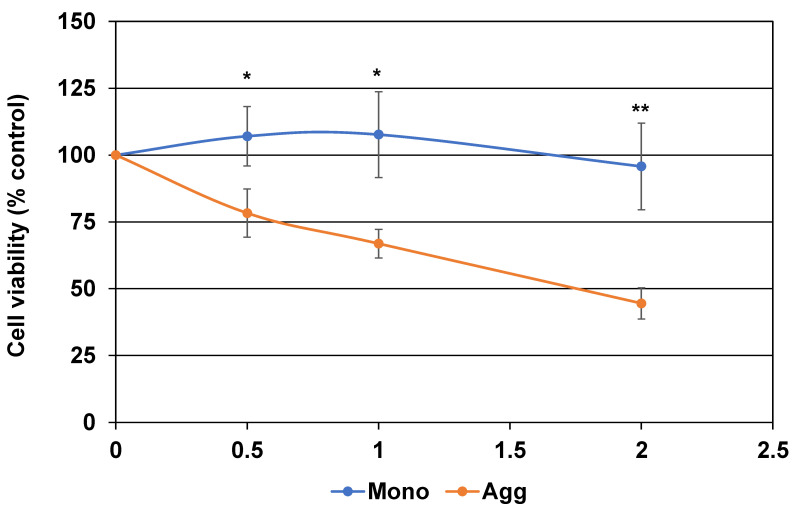
Effects of CST3 aggregation on the viability of a human astrocyte cell line. A human astrocyte cell line culture was treated with indicated concentrations of non-aggregated and aggregated recombinant CST3 for 48 h. Cell viability after incubation was evaluated by MTT assay, as described in the Materials and Methods. The data was calculated as %control, where cells cultured without CST3 were served as such, and expressed as mean ± SD of at least three independent experiments. Mono = CST3 monomer, Agg = CST3 aggregated at 37 °C. Statistical significance is denoted as follows; * *p* < 0.05 vs. corresponding aggregated CST3, ** *p* < 0.005 vs. corresponding aggregated CST3 condition.

*Effects of CST3 aggregation on cell viability:* Finally, we checked the effects of CST3 aggregation on the viability of CCF-STTG1 in culture by MTT assay. The result showed that the aggregated form of CST3 dose-dependently inhibited the viability of CCF-STTG1 cells in culture, whereas the monomeric form of CST3 did not show such effects (Figure 6).

## 3. Discussion

In this study we demonstrated that after aggregation, CST3 lost its protease inhibitory activity at physiological pH (7.4) and at pH that is near the lysosomal environment (5.5). It is known that in cerebral and peripheral arteries, CST3 deficiency can induce aneurysmal changes [24,25,26,27]. Such changes are largely due to the unchecked activities of proteases such as cathepsins. Consequently, extensive changes of connective tissues and cells of the vessels are seen. In addition, the precise balance of CST3 and proteases is essential for vessel remodeling, and polymorphisms in the gene that alter CST3 levels are found to be associated with pathology such as cerebral small vessel disease [23,28,29,30]. Aggregated CST3 has a diminished capability to inhibit cathepsin B activity, indicating its reduced function. Moreover, aggregated protein, especially immature aggregates, can interact with cell membranes and make ion channels, leading to the destabilization of the internal environment of the cells [31]. Taken together, the deposition of such aggregated CST3 in the vessel walls during CAA can play an important role in the pathology by altering protease activities and cellular viability. In addition to the vessels, these soluble immature aggregates could also interact with neighboring cells, including neurons and astrocytes. Such interaction of toxic CST3 aggregates could be an independent reason for cellular degeneration, as implied by our cell culture studies.

In CAA, CST3 has been shown to be deposited in the vessel wall along with Aβ peptide [19]. The ratio of Aβ and CST3 in the lesion area is about 100:1 [19]. Hence, Aβ is the primary deposit here, and low level CST3 might try to modulate the course of deposition. A previous study has demonstrated that CST3 can inhibit Aβ aggregation after binding with the peptide [17], indicating a protective role in the pathology. Since aggregated CST3 did not inhibit Aβ fibril formation, rather, slightly increasing its levels, such species of the protein could lose its protective effects, resulting in an unchecked deposition of Aβ. Moreover, a study showed that in the cases of CAA with cerebral hemorrhage, CST3 is most likely to be co-deposited with Aβ [32], indicating a causal relationship between them. Aβ used to be deposited in the entire vascular wall, whereas CST3 is positive in the outer media and adventitia layers [32,33]. Similar areas of cathepsins are shown to be positive in cerebral aneurysms [34]. Thus, it is possible that CST3 is deposited in aggregated form, resulting in loss of protease inhibitory activity. Then, combining the effects of unchecked protease activity and Aβ toxicity cause excessive damage to the vessel wall seen in CAA.

A previous study showed that the oligomeric form of CST3 retains the inhibitory properties of Aβ aggregation and cathepsin activities [35]. The reason for this discrepancy with our results could be the difference of the preparation procedure of CST3 oligomers. In that study, CST3 samples contain some oligomers, which were separated using membrane filters. Such oligomers were produced by non-domain swapping aggregation and retain their inhibitory properties. A three-dimensional structure analysis showed that CST3 consists of five anti-parallel β-sheets wrapped around a long α-helix. β-sheet 2 and 3 are connected to each other with hairpin loop1 (L1) and β-sheet f 4 and 5 with hairpin loop2 (L2). The L1, L2, and N terminal amino acids of CST3 align together and form the motif that inhibits C1 type protease activities [1]. During aggregation by domain swapping, the CST3 monomer unfolded partially, then combined with another molecule by exchanging subdomains, resulting in dimer and other higher order aggregates [1,36]. During the process of oligomerization, the L1 loop present in the canonical CST3 three-dimensional structure disappears, which results in a loss of C1 protease inhibitory activities [1,22,37]. In our aggregation process, prolonged incubation with agitation at 37 °C could induce a partial unfolding and, consequently, aggregation by domain swapping, which results in loss of cathepsin inhibition properties.

Physical conditions such as heat can alter the aggregation status of protein [38]. At increased temperature, protein adopts an unfolded conformation before being aggregated [38,39]. It is shown that some proteins are aggregation-prone, even in mild heat stress conditions [40]. In this study, we found that CST3 can be aggregated during prolonged incubation at physiological temperature with sufficient agitation, suggesting that CST3 could be an aggregation-prone protein [41]. CST3 can also be aggregated at high temperatures (80 °C). However, it did not adopt an oligomeric structure at this temperature. Hence, it might adopt an amorphous conformation in this condition [42]. In addition, 80 °C is far above the physiological temperature. Thus, we did not pursue further with this temperature. At 37 °C, CST3 clearly showed various sizes of oligomeric species. In addition, some aggregates adopt fibrillary structure, indicating the aggregation-prone properties of CST3.

In culture conditions, including a neuroblastoma cell culture [43,44], CST3 is demonstrated to internalize and localize in the lysosome. In those studies, the monomeric form of CST3 was used to see the internalization. Our study demonstrated that the aggregated form of CST3 can also be internalized more efficiently than its monomeric form. Although we did not investigate the process of internalization, the possible mechanism could be endocytosis, because the size of the aggregates was quite big, as revealed by the electron microscopy. Most of the proteins associated with neurodegenerative diseases have the propensity to make fibrillar aggregate. Such fibrils have the ability to infiltrate into the cells through endocytosis [45]. For endocytosis, several points including membrane properties, membrane receptor-mediated binding, and the monomeric and fibrillar structures of the proteins have been proposed to be important [42,43,44,45,46]. Although we did not elucidate the exact mechanisms, it is possible that aggregated CST3 interacts with cell membranes more robustly than its monomeric form, causing the endocytic internalization efficient. The internalized proteins were found to be distributed in a dot-like pattern, indicating its location in the lysosome. In a previous study, we demonstrated the localization of CST3 in neuronal lysosomes in culture condition and in human brains (20). Hence, the accumulation of aggregated CST3 might alter the lysosomal activities, which could be detrimental for the cells. Indeed, we found that aggregated CST3 is toxic to the cells in culture.

Our study provides an important insight into the functional changes of CST3 after aggregation. However, some limitations of the study should be noted. First, our study used an in vitro system to analyze the functional properties of CST3, not animal models or pathological specimens. Since animal models and pathological conditions are far more complex than in vitro systems [47], it will be worthwhile to characterize the deposited CST3 in the animal models and pathological conditions such as CAA in respect of structural and functional changes. Second, our study showed that physiological temperature is enough to induce aggregation, even stored at cold conditions can induce its aggregation [48]. Hence, some factors might be involved in vivo conditions that keep CST3 in monomeric form. Identification of such factors could be important for a better understanding of the pathology of CST3-related diseases such as CAA and the subsequent identification of potential therapeutic targets for the disease.

## 4. Conclusions

In conclusion, CST3 aggregated in physiological temperature has altered properties that reduced its protective effects on protease activities and Aβ aggregation. Such altered properties of CST3 could be important in the pathophysiology of CAA and neurodegenerative diseases such as ALS.

## 5. Materials and Methods

### 5.1. Preparation of Recombinant CST3

To prepare recombinant protein, a bacterial expression vector (CST3-pQE32) was generated by inserting the entire coding region of CST3 into a pQE32 expression vector containing His6 tag (Qiagen, Hilden, Germany). Schematic representation of vector generation is shown in Supplemental Figure S1. To generate the vector, the coding sequence of CST3 was PCR amplified using Phusion High-Fidelity DNA polymerase (ThermoFisher, Waltham, MA, USA), where human brain cDNA (Clontech, Mountain View, CA, USA) was used as a template. In addition to cDNA sequence, forward primer contained a SphI and that of reverse primer a HindIII sites at their 5′ ends. Then, the PCR product and pQE32 vector were digested with SphI and HindIII (New England BioLab, Ipswich, MA, USA). Digested PCR product ligated with digested pQE32 vector using Ligation High enzyme kit (Toyobo, Osaka, Japan) to prepare CST3-pQE32 vector. The vector was used to transform chemically competent Escherichia coli bacteria (JM109; Promega, Madison, WI, USA). Then, CST3 were expressed in transformed JM109 by 0.4 mM Isopropyl β-D-1-thiogalactopyranoside (IPTG) and purified by affinity chromatography using Ni-NTA Superflow columns (Qiagen) according to the manufacturer’s instructions.

### 5.2. Aggregation of CST3

To induce aggregation, recombinant CST3 monomer dissolved in PBS (pH 7.4) (1.3 mg/mL) was incubated at 37 °C with vigorous agitation or at 80 °C without agitation for indicated times.

### 5.3. Aggregation Assay

Aggregation of the proteins were analyzed using a kit (PROTEOSTAT aggregation assay, Enzo Life Sciences, Farmingdale, NY, USA) according to the manufacturer’s instruction. Briefly, 2 µL of diluted PROTEOSTAT detection reagent was dispensed at the bottom of a well of a 96-well plate. After incubation of the protein at indicated conditions for indicated times, 98 µL of the protein was taken from the solution and directly added to the well and incubated at room temperature for 15 min in the dark. Then, the generated signal was read using a fluorescence microplate reader with excitation and emission wavelengths at 550 and 600 nm, respectively. Along with the test samples, both positive and negative controls, as well as blanks (1× assay buffer alone), were analyzed. The data are presented as the average ± standard deviation (SD) of an arbitrary fluorescence unit.

### 5.4. Dot-Blot Oligomer Assay

After aggregation, 2.6 µg of the proteins were spotted on a nitrocellulose membrane. As a negative control, CST3 monomer samples were used. The membrane was incubated in a blocking solution (2% bovine serum albumin in PBS, 0.5% Tween20) for 1 h. Then, the membrane was blotted with an oligomer-specific antibody (A11, Invitrogen, Waltham, MA, USA, rabbit, 1:1000) diluted in blocking solution for 2 h at room temperature. This antibody specifically reacts with a variety of soluble oligomeric protein aggregates regardless of their amino acid sequence and does not react with monomer species or insoluble fibrils of protein/peptide [49]. To detect immunoreactive protein, an infrared dye conjugated anti-rabbit IgG (Li-COR Biosciences, Lincoln, NE, USA, diluted 5000 times with blocking solution) was added to the membrane, incubated for 1 h at room temperature, and scanned with Odyssey infrared dye scanning system (Li-COR), according to the manufacturer’s instructions. After every incubation, the membrane was washed with a wash buffer (0.5% Tween20 in PBS), except after blocking. After stripping, the membrane was also blotted with anti-CST3 antibody (Abcam, Waltham, MA, USA, rabbit, 1:1000), which served as a loading control.

### 5.5. Electron Microscopy

Electron microscopic analysis of monomeric and aggregated forms of CST3 was performed as described previously [50]. In a brief, 5 μL of monomeric or aggregated CST3 (1.3 mg/mL) was applied to a carbon coated Formvar grid (Nisshin EM Co., Tokyo, Japan) and incubated for 1 min. After incubation, 1 drop of 0.5% *v*/*v* glutaraldehyde solution was applied to the grid and incubated again for 1 min. Then, the grid was washed with a few drops of water and dried. Ten microliters of 2% *w*/*v* uranyl acetate solution were applied to the dried grid and incubated for 2 min. Finally, excess uranyl acetate was soaked with a paper towel, and the grid was air-dried and examined under an EM–002B electron microscope (Topcon, Tokyo, Japan).

### 5.6. Western Blotting

Western blotting was performed as described previously [51]. In a brief, equal volume of non-reducing Western blotting sample buffer (125 mM Tris HCl, pH 6.8; 20% glycerol; 4% SDS; 0.1% bromophenol blue) was added to monomeric and aggregated CST3, and incubated at 85 °C for 2 min. Then 2 μg equivalent of monomeric and aggregated proteins were separated by electrophoresis using 15% polyacrylamide gel. After transfer to a PVDF membrane, the membrane was incubated with anti-CST3 IgG (rabbit, Abcam). The Immunoreaction was identified using infrared-fluorophore-conjugated anti-rabbit IgG (Li-COR, diluted 5000 times with blocking solution), and the signals of Immunoreactive proteins were detected using an infrared scanner (Li-COR).

### 5.7. Cathepsin B Assay

Cathepsin B activity was measured, as described previously [20]. In a brief, 75 µL reaction mixture was prepared by adding cathepsin B (2.5 µg/mL, 10 µL), and indicated concentrations of aggregated or monomeric CST3 to a reaction buffer (final concentrations: 20 mM L-cysteine and 0.13 mM sodium acetate, pH 5.5 or 7.4), mixed and incubated for 5 min at 37 °C. Then, 20 µL of substrate (Z-Arg-Arg-MCA; 20 μM, Peptide Institute, Osaka, Japan) was added to the reaction mixtures and incubated for another 1 h at 37 °C. The enzymatic reaction was terminated with 100 µL of 0.1 M monochloroacetate (pH 4.3), and the levels of released MCA was measured with a multimode microplate reader (DTX880; Beckman coulter, Brea, CA, USA), with excitation and emission wavelengths at 370 nm and 465 nm, respectively.

### 5.8. Aβ_1-40_ Peptide Fibril Formation

For fibril formation, 50 µL solution of synthetic Aβ_1-40_ peptide (Peptide Institute, Osaka, Japan) (25 μM) in fibril formation buffer (50 mM phosphate buffer pH 7.5 and 100 mM NaCl) was prepared with or without CST3 monomer or aggregates (1.3 µg). The reaction mixture was incubated at 37 °C without agitation for 48 h, and then the fibril formation reaction was terminated by quickly freezing the samples.

### 5.9. Assessment of Fibril Levels Using ThT Fluorescence

After incubation, the presence of β-sheet structures in the reaction mixtures were monitored by means of ThT fluorescence spectroscopy. Samples were diluted tenfold with glycine (pH 8.5, 50 mM final concentration) and ThT (5 μM final concentration). ThT fluorescence was measured using a fluorescence spectrophotometer (F2500 spectrofluorometer, Hitachi, Tokyo, Japan), with excitation and emission wavelengths of 446 and 490 nm, respectively. The normalized florescence intensities of the β-sheet amyloids were obtained by subtracting the florescence intensity of buffer alone from that of the sample.

### 5.10. Cell Culture

#### 5.10.1. CCF-STTG1 Culture

A human astrocytoma cell line (CCF-STTG1) was obtained from American Type Culture Collection (ATCC, Manassas, VA, USA) and cultured in RPMI-1640 medium (ATCC) containing 10% fetal bovine serum (FBS, Gibco, Invitrogen). During stimulations with aggregated or monomeric CST3, FBS concentration was reduced to 1%.

#### 5.10.2. Culture of a Mouse Neuronal Stem Cell Line and Differentiation to Mature Neurons

Neuronal stem cells were isolated from fetus of a wild-type mouse with MRL background at 14.5 days of gestation and a neuronal stem cell line (NMW7) was generated, as described previously [52]. The cell line shows similar morphological, expressional, and differentiation potentials as primary mouse neuronal stem cells. NMW7 was plated in a tissue culture dish in complete media (high glucose DMEM: F12 ham 1:1, 2% FBS, N2 supplement, bFGF 20 ng/mL, and EGF 20 ng/mL). For differentiation, neurospheres were generated by culturing the cells in neurosphere medium (high glucose DMEM: F12 ham 1:1, bFGF 20 ng/mL, EGF 20 ng/mL, N2 supplement, and B27 supplement) for 3 days. Neurospheres containing medium were transferred to poly-L-lysine (PL)-coated dishes, and an equal amount of differentiation medium (DMEM: F12 ham 1:1, B27) was added and cultured. When the neurospheres were attached, the medium was removed; fresh differentiation medium was added and cultured for another 14 days. After differentiation, indicated concentrations of non-aggregated or aggregated recombinant CST3 was added to the medium, and the culture was continued for another 4 days.

### 5.11. (3-(4,5-Dimethylthiazol-2-yl)-2,5-diphenyltetrazolium Bromide (MTT) Assay

The toxicity of monomeric and aggregated CST3 was evaluated by an MTT cell viability assay, as described previously [24]. Briefly, CCF-STTG1 cells (3 × 10^3^/well) were seeded into wells of a 96-well plate and cultured for 24 h. The cells were treated with the indicated concentrations of monomeric or aggregated CST3 in 100 μL DMEM containing 0.5% FBS for 48 h. After incubation, 20 μL of MTT solution (Sigma Aldrich, St. Louis, MO, USA) (5 mg/mL) was added to the culture medium, and incubation was continued for 3.5 h at 37 °C. Then, the medium was removed carefully, MTT solvent (4 mM HCl, 0.1% Nonidet-P-40 in isopropanol, 150 µL) was added to the wells, and the plate was incubated at room temperature for 15 min with protection from light. Then, the absorbance was read at 590 nm. The absorbance of the cells culture without CST3 were used as a control.

### 5.12. Immunocytochemistry

For immunocytochemistry, cells were cultured in appropriate 8-well chamber slides. After culture, CCF-STTG1 or NMW7 cells were fixed with 4% paraformaldehyde in phosphate-buffered saline (PBS) for 15 min and blocked with 5% normal goat serum and 0.1% Triton X-100 in PBS for 30 min. Then, anti-His6 IgG (mouse, 1:50; Roche, Basel, Switzerland) or anti-CST3 IgG (rabbit, 1:200; Abcam) was added to the wells and incubated overnight at 4 °C. Immunoreactive proteins were detected with Texas Red-conjugated goat anti-rabbit IgG (1:200; Santa Cruz Biotechnology, Santa Cruz, CA, USA) or FITC-conjugated goat anti-mouse IgG (1:200; Santa Cruz Biotechnology). The fluorescence signals were visualized using a fluorescence microscope equipped with filters for individual fluorophores (FITC emission filter wavelength 525 nm, Texas red emission filter wavelength 620 nm, Hoechst emission filter wavelength 460 nm) (ECLIPSE E600, NIKON, Tokyo, Japan).

### 5.13. Statistical Analysis

Numerical data are expressed as means ± SD of at least three independent experiments. Statistical analysis to compare mean values was performed using one-way ANOVA, followed by Scheffe’s post hoc test or Student’s T TEST, and *p* value < 0.05 was considered as statistically significant.

## Data Availability

All data of this study are shown in the report.

## Supplementary Materials

The following are available online at https://www.mdpi.com/article/10.3390/ijms22189682/s1, Figure S1: Human CST3 coding sequence and schematic design of CST3 expression vector generation; Table S1: CST3 aggregation assay; Table S2: Cathepsin B inhibition assay (pH 5.5); Table S3: Cathepsin B inhibition assay (pH 7.4); Table S4: Effects of CST3 aggregation on Aβ1-40 fibril formation; Table S5: Effects of CST3 aggregation on the viability of a human astrocyte cell line.
