# Supplementary material for: Aggregation of Cystatin C Changes Its Inhibitory Functions on Protease Activities and Amyloid β Fibril Formation
Source: Int J Mol Sci. 2021 Sep 7;22(18):9682. doi: 10.3390/ijms22189682 (PMC8465189; doi:10.3390/ijms22189682)
Supplement: Supplementary file 1 [file ijms-22-09682-s001.zip › ijms-1339134-supplementary.pdf]

International Journal of
Molecular Sciences

MDPI

Article

Aggregation of Cystatin C Changes Its Inhibitory Functions on Protease Activities and Amyloid β Fibril Formation

Abdullah Md. Sheikh [1,†], Yasuko Wada [2,†], Shatera Tabassum [1], Satoshi Inagaki [2], Shingo Mitaki [2], Shozo Yano [1] and Atsushi Nagai [1,2,*]

1 Department of Laboratory Medicine, Faculty of Medicine, Shimane University, 89-1 Enya Cho, Izumo 693-8501, Japan; abdullah@med.shimane-u.ac.jp (A.M.S.); tabassum@med.shimane-u.ac.jp (S.T.); syano@med.shimane-u.ac.jp (S.Y.)

2 Department of Neurology, Faculty of Medicine, Shimane University, 89-1 Enya Cho, Izumo 693-8501, Japan; wadayasu@med.shimane-u.ac.jp (Y.W.); inasato061009@yahool.co.jp (S.I.); shingomi@med.shimane-u.ac.jp (S.M.)

* Correspondence: anagai@med.shimane-u.ac.jp; Tel./Fax: +81-0853-20-2198

† Authors contributed equally to this work.

Supplemental information

Supplemental tables:

Table S1. CST3 aggregation assay.

	0 h	80°C plateau	37°C plateau
Fluorescence intensity (arbitrary unit)	176350.67 ± 7353.8	1165175.33 ± 623076.6[a, b]	395962.63 ± 25122.2[b]

CST3 aggregation at the plateau after incubation at 80°C or 37°C were measured using an aggregation assay kit, where the fluorescence intensities (arbitrary unit) represented the concentrations of the aggregated protein in the samples. The results presented here as average ± SD of at least 3 independent experiments. Statistical significance is denoted as follows; [a]$p < 0.05$ vs 37°C plateau condition, [b]$p < 0.01$ vs 0 h condition.

Table S2. Cathepsin B inhibition assay (pH 5.5).

CST3 (pmol/L)	Monomer	Aggregate
0	98.8 ± 1.2	98.8 ± 1.2
5	92.8 ± 6	107.2 ± 0.9[a]
10	82.3 ± 11.5	106.6 ± 3.8[a]
20	79.1 ± 4	98.1 ± 6.6[a]
50	37.8 ± 1.7	76.5 ± 8.4[b]

Indicated concentrations of CST3 monomers, or CST3 aggregated at 37°C was used to investigate their capabilities to inhibit cathepsin B activitiy at pH 5.5. The results were calculated as %calibrator, where one sample of cathepsin B activity without CST3 was served as such, and presented here as average ± SD of at least 3 independent experiments.

Statistical significance is denoted as follows, $^{a}p < 0.01$ vs corresponding CST3 monomer, and $^{b}p < 0.001$ vs corresponding CST3 monomer conditions.

Table S3. Cathepsin B inhibition assay (pH 7.4).

CST3 (pmol/L)	Monomer	Aggregate
0	98.8 ± 2.4	98.8 ± 2.4
1	93.1 ± 0.6	99.1 ± 6.6
2	75.4 ± 11.1	98.1 ± 3.7[a]
5	41.5 ± 0.6	102.5 ± 1.6[b]
10	19.3 ± 7.4	90.2 ± 7.4[b]
20	14.7 ± 1.9	96.3 ± 0.9[b]

Indicated concentrations of CST3 monomers, or CST3 aggregated at 37°C was used to investigate their capabilities to inhibit cathepsin B activitiy at pH 7.4. The results were calculated as %calibrator, where one sample of cathepsin B activity without CST3 was served as such, and presented here as average ± SD of at least 3 independent experiments. Statistical significance is denoted as follows, $^{a}p < 0.01$ vs corresponding CST3 monomer, and $^{b}p < 0.001$ vs corresponding CST3 monomer conditions.

Table S4. Effects of CST3 aggregation on Aβ_{1-40} fibril formation.

	CST3 monomer	CST3 oligomer	Aβ_{1-40}	Aβ_{1-40} + CST3 monomer	Aβ_{1-40} + CST3 oligomer
ThT fluorescence intensities	0.06 ± 0.01	0.12 ± 0.05	7.2 ± 2.5	4.05 ± 1.6[b]	8.7 ± 1.3[a, c]

Aβ_{1-40} (25 μM) was incubated in the absence or presence of non-aggregated or aggregated (at 37°C) CST3 (1.3 μg/ 50 μL) for 48 h. Amyloid fibrils formed after incubation was measured by ThT fluorescence assay. Average fluorescence emission values are shown here, and the data are presented as the mean ± SD of at least 3 independent experiments. Statistical significance is denoted as follows; $^{a}p < 0.05$ vs Aβ_{1-40}, $^{b}p < 0.005$ vs Aβ_{1-40}, $^{c}p < 0.0001$ vs Aβ_{1-40} and CST3 monomer condition.

Table S5. Effects of CST3 aggregation on the viability of a human astrocyte cell line.

CST3 (pmol)	Monomer	Aggregate
0	100	100
0.5	107.04 ± 11.09	78.28 ± 9.04[a]
1	107.65 ± 16.09	66.86 ± 5.37[a]
2	95.77 ± 16.21	44.51 ± 5.86[b]

A human astrocyte cell line culture was treated with indicated concentrations of non-aggregated and aggregated recombinant CST3 for 48 h. Cell viability after incubation was evaluated by MTT assay, as described in the Materials and Methods. The data was calculated as %control, where samples without CST3 conditions were served as such, and presented as the mean ± SD of at least 3 independent experiments. Statistical significance is denoted as follows; $^{a}p < 0.05$ vs corresponding aggregated CST3, $^{b}p < 0.005$ vs corresponding aggregated CST3 condition.

Supplemental figure:

(A)

Human CST3 coding sequence

atg gcc ggg ccc ctg cgc gcc ccg ctg ctc ctg ctg gcc atc ctg gcc gtg gcc ctg gcc gtg agc
ccc gcg gcc ggc tcc agt ccc ggc aag ccg ccg cgc cta gtg gga ggc ccc atg gac gcc agc gtg
gag gag gag ggt gtg cgg cgt gca ctg gac ttt gcc gtc ggc gag tac aac aaa gcc agc aac gac
atg tac cac agc cgc gcg ctg cag gtg gtg cgc gcc cgc aag cag atc gta gct ggg gtg aac tac
ttc ttg gac gtg gag ctg ggc cga acc acg tgt acc aag acc cag ccc aac ttg gac aac tgc ccc
ttc cat gac cag cca cat ctg aaa agg aaa gca ttc tgc tct ttc cag atc tac gct gtg cct tgg cag
ggc aca atg acc ttg tcg aaa tcc acc tgt cag gac **gcc**

(B)

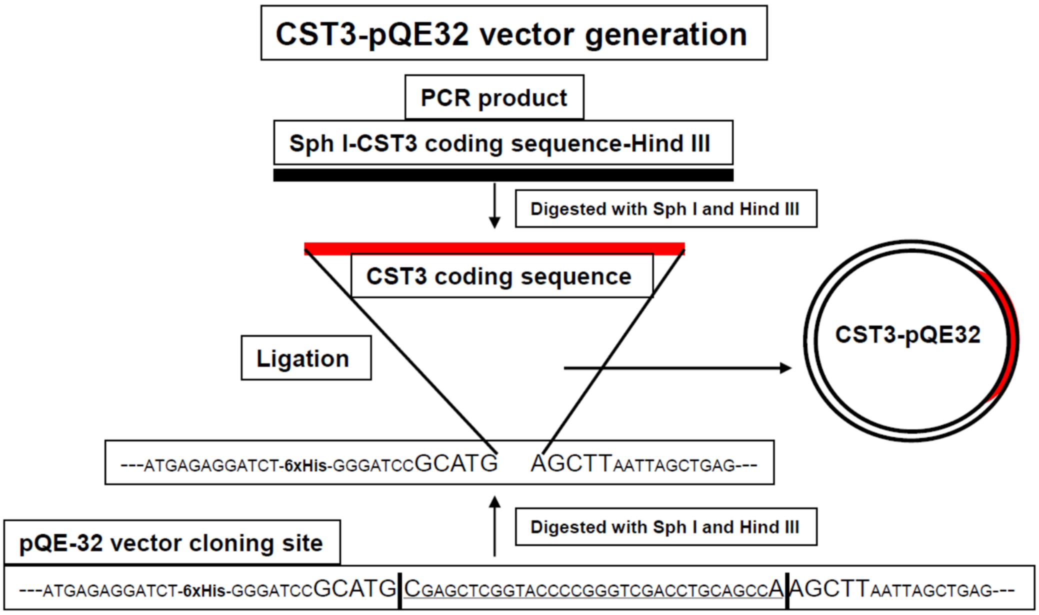

Figure S1. Human CST3 coding sequence and schematic design of CST3 expression vector generation: To prepare recombinant CST3 protein, a bacterial expression vector was generated. The entire coding sequence human CST3 was PCR amplified and inserted into a bacterial expression vector (pQE32), as described in the Materials and Methods. (A) showed the coding sequence of human CST3 published in NCBI (NM_000099.4). In (B), the schematic representation that depict the method of generation of human CST3 expressing bacterial vector are shown. GCATGC is SphI restriction enzyme site, where the enzyme cut the DNA between G and C, as indicated. AAGCTT is HindIII restriction enzyme site, where the enzyme cut the DNA between A and A, as indicated.
